# Elevated free fatty acid level is a risk factor for early postoperative hypoxemia after on-pump coronary artery bypass grafting: association with endothelial activation

**DOI:** 10.1186/s13019-015-0323-9

**Published:** 2015-09-17

**Authors:** Sheng Shi, Yuan Gao, Limin Wang, Jian Liu, Zhongxiang Yuan, Min Yu

**Affiliations:** 1 Department of Cardiovascular Surgery, Shanghai First People’s Hospital, Shanghai Jiao Tong University School of Medicine, No. 100 Haining Road, Shanghai, 200080 P.R. China; 2 Department of Cardiovascular Surgery, Taian City Central Hospital, 29 Longtan Road, Taian, Shandong Province 271000 P.R. China

**Keywords:** Free fatty acid, Coronary artery bypass grafting, Hypoxemia, Cardio- pulmonary bypass, Mechanism

## Abstract

**Background:**

We aimed to investigate the relationship between increased free fatty acid (FFA) level and early postoperative hypoxemia after coronary artery bypass grafting (CABG) with cardiopulmonary bypass (CPB).

**Methods:**

Ninety-eight consecutive patients undergoing CABG were enrolled. Early postoperative hypoxemia was defined as the lowest of the ratio of arterial oxygen tension (PaO_2_) to inspired oxygen fraction (FiO_2_) ≤ 200 mm Hg within 24 h without pleural effusion and pneumothorax. The 26 perioperative factors, serum levels of FFA and inflammatory cytokines between the hypoxemia and non-hypoxemia groups were recorded or detected using autoanalyzer and enzyme-linked immunosorbent assay, respectively. Additionally, the risk factors for early postoperative hypoxemia were evaluated using multiple logistic regression analysis.

**Results:**

The incidence rate of early postoperative hypoxemia was 37.8 %. Serum FFA levels were significantly higher in the hypoxemia group than in the non-hypoxemia group (*P* < 0.001). Further, postoperative serum FFA levels were inversely related to the lowest of the ratio of PaO_2_/FiO_2_ at 24 h after CABG (r = − 0.367, *P* < 0.001). Multiple logistic regression analysis confirmed that age, body mass index and postoperative serum FFA concentrations were independently associated with early postoperative hypoxemia. Notably, patients with hypoxemia had markedly higher serum intercellular adhesion molecule-1 (ICAM-1) levels than those without (*P* < 0.001). Moreover, serum FFA levels at 2 h after CABG correlated positively with ICAM-1 concentrations (r = 0.492, *P* < 0.001).

**Conclusions:**

Elevated FFA concentration is a risk factor for early postoperative hypoxemia after on-pump CABG, which may be closely associated with endothelial activation.

## Background

Coronary artery bypass surgery has traditionally been performed with cardiopulmonary bypass (CPB). Coronary artery bypass grafting (CABG) with CPB could result in systemic inflammatory response, lung ischemia-reperfusion injury, blood transfusion and surgery procedure and further cause a series of pathophysiological changes of lung injury including pulmonary edema, decreased lung compliance, disturbance of ventilation-perfusion ratio and increased pulmonary vascular resistance, finally leading to postoperative hypoxemia [1–3]. Postoperative hypoxemia will extend the stays in intensive care unit (ICU) and hospital, and increase various complications as well as mortality [2]. Therefore, an understanding of risk factors and mechanisms may help to prevent hypoxemia after cardiac surgery and improve patient outcomes [2,3].

Free fatty acid (FFA) is a kind of long-chain fatty acids produced by triacylglycerol hydrolysis. A total of 37 different types of FFAs have been detected in human serum [4]. A high-dose heparin was used to anticoagulate the perfusion circuit during hypothermic CPB [5]; and it could hydrolyze triglycerides (TG) and increase serum FFA levels [6–8]. Local increased FFA is the cause of lung injury secondary to pulmonary fat embolism [9]. Animal experiments of hypertriglyceridemia demonstrated that the addition of heparin could result in high FFA level and lung injury such as pulmonary edema, intrapulmonary shunt, and increased pulmonary vascular resistance [7]. Furthermore, the calculated ratios of serum FFAs (the ratio of C18 unsaturated fatty acids linoleate and oleate to fully saturated palmitate) increased and predicted the development of acute respiratory distress syndrome (ARDS) in at-risk patients with sepsis and/or trauma [10]. During CPB, chylomicron could result in elevated levels of serum FFA, which was associated with ARDS and ischemic brain injury [11].

However, the relationship between increased FFA level and early postoperative hypoxemia after CPB remains not completely understood. In this study, we hypothesized that elevated FFA concentration in cardiac surgery with CPB was involved in the development of lung injury and was a risk factor of early postoperative hypoxemia. The aim of this study was to demonstrate the hypothesis, and to explore the possible mechanisms.

## Methods

### Subject

From March to December 2012, 112 consecutive patients undergoing CABG in our hospital were enrolled. Exclusion criteria included preoperative hypoxemia [arterial oxygen tension (PaO_2_) < 80 mm Hg with oxygen inhalation via nasal cannula], chronic obstructive pulmonary disease, asthma, hepatic and renal failure, acute left heart failure, acute myocardial infarction, emergency surgery, low cardiac output syndrome in 24 h after cardiac surgery, and serious complications of liver, kidney and brain. Twelve were excluded, and 2 patients’s blood samples were insufficient for analysis. Ninety eight patients were eligible for the present study. Early postoperative hypoxemia was defined as the lowest of the ratio of PaO_2_ to inspired oxygen fraction (PaO_2_/FiO_2_) ≤ 200 mm Hg within 24 h without pleural effusion and pneumothorax [3]. The patients were divided into two groups by early postoperative PaO_2_/FiO_2_: hypoxemia group and non-hypoxemia group. The 26 perioperative factors between the two groups were compared. The study was approved by the Institutional Medical Ethics Committee of Shanghai First People’ s Hospital affiliated to Shanghai Jiaotong University reviewed all research and was consistent with the Declaration of Helsinki. All patients gave written consent.

### Method

The operation was performed in inhalation-intravenous general anesthesia. Anesthesia was induced with midazolam (2–3 mg), fentanyl (0.2 mg), propofol (0.5–1.5 mg/kg) and vecuronium, and maintained by isoflurane as well as continuous infusion of propofol (2–5 mg/kg/h). Fentanyl (0.1–0.2 mg) was intravenously administered before skin incision, with total amount of fentanyl less than 15 μg/kg during operation. CABG was carried out through median sternotomy using standard CPB with single venous right atrial and ascending aortic cannulation. Moderate systemic hypothermia (28 °C) was applied and CBP was carried out with disposable membrane oxygenator (Medtronic, AFFINITY NT, Medtronic. Inc. USA) and centrifugal pump (Stockert III, Germany). Myocardial preservation was achieved with aortic root infusion of mild blood cardioplegia, with repetition every 20 min both from the aortic root and saphenous veins during the cross-clamp period. A blood gas analyzer (ABL-80 FLEX, Radiometer, Copenhagen, Denmark) was used to measure arterial partial pressure of carbon dioxide (PaCO_2_) and PaO_2_ perioperatively. All postoperative patients were ventilated by Drager Savina respirator (Drägerwerk AG & Co. KGaA, Lübeck, Germany).

A 5 ml venous blood sample for each patient was collected at 4 different time points: preoperation, 10 min after heparinization, after protamine neutralization, and 2 h postoperation, respectively. Plasma was separated in 10 min after heparinization, whereas serum was obtained at the rest 3 different time points. All blood samples were first stored at −80 °C and then thawed as well as assayed for TG, total cholesterol (TC), FFA using an autoanalyzer (ADVIA2400 Chemistry System, Siemens Healthcare Diagnostics Ltd, Germany). Serum samples of 2 h postoperation were collected to detect inflammatory cytokines including interleukin-6 (IL-6), endothelin-1 (ET-1), tumor necrosis factor-α (TNF-α), and intercellular adhesion molecule-1 (ICAM-1), using commercial enzyme-linked immunosorbent assay kits (Zhili Biotechnology,Shanghai, China) according to the manufacturer’s instructions. PaO_2_ was measured at 1 h, 2 h, 6 h, 12 h and 24 h after termination of operation. Perioperative variables were obtained from patients’ medical records.

### Statistical analyses

Data management and analysis were performed with SPSS 17.0 (SPSS, Chicago, IL). Continuous variables were expressed as mean ± standard derivation and performed normality test. Data with normal distribution was analyzed by unpaired *t* test, and data with nonnormal distribution was by Komolgorov-Smirnov Test. Categorical variables were expressed as percentages and assessed by chi-square test. Pearson correlations were analyzed to determine the relationship among FFA, IL-6, ET-1, TNF-α, ICAM-1, and lowest PaO_2_/FiO_2_. Polynomial regression analysis was used to detect the relationship between FFA and TG levels, FFA and the lowest PaO_2_/FiO_2,_ FFA and ICAM, ICAM and the lowest PaO_2_/FiO_2_. Multiple stepwise logistic regression anlysis was used to identify independent risk factors for early postoperative hypoxemia. A *P* value of < 0.05 was considered statistically significant.

## Results

### Clinical characteristics of perioperative patients

98 patients were eligible for the present study (72 males and 26 females, mean age 61.69 ± 9.29 years, ranging from 45 to 78 years), and 37 developed early postoperative hypoxemia, accounting for 37.8 %. Perioperative characteristics of all subjects are shown in Table 1. There were more elder patients in the hypoxemia group than in the non-hypoxemia group (65.54 ± 10.11 *vs.* 59.36 ± 7.97 years, *P* = 0.002). Compared with the non-hypoxemia group, patients in the hypoxemia group had more male (86.5 % *vs* 65.6 %, *P* =0.033), markedly higher body mass index (BMI, 26.45 ± 2.77 *vs.* 23.98 ± 2.92, *P* < 0.001), serum TC levels (4.64 ± 1.05 *vs.* 4.14 ± 1.05 mmol/l, *P* = 0.016), number of graftings (2.70 ± 0.94 *vs.* 2.08 ± 0.73, *P* = 0.001), and serum FFA concentrations (0.87 ± 0.28 *vs.* 0.66 ± 0.22 mmol/l, *P* < 0.001). The time of CPB and aortic clamping in the hypoxemia group were much longer than those in the non-hypoxemia group (103.73 ± 37.01 *vs.* 79.26 ± 28.10 min, *P* < 0.001; 64.05 ± 23.31 *vs.*51.73 ± 64.05 min, *P* = 0.01). Patients of postoperative hypoxemia had more volume of transfusion in comparison with those of non-hypoxemia (729.73 ± 790.52 *vs.* 277.05 ± 382.16 ml, *P* < 0.001). There were no significant differences in other clinical characteristics such as preoperative hypertension, left ventricular ejection fraction (LVEF), serum TG levels and the time of incubation and hospitalization between the hypoxemia and non-hypoxemia groups. ICU stay was longer for patients of hypoxemia (3.65 ± 2.50 vs 2.59 ± 2.59,p = 0.027). Although a patient in the hypoxemia group died from ventricular fibrillation at day 3 after CABG and all subjects in the non-hypoxemia group survived until discharge, there was no statistically different mortality between the two groups.

**Table 1:** Clinical characteristics of perioperative patients

	Hypoxemia group	Non-hypoxemia group	*P* value
	(*n* = 37)	(*n* = 61)	
Preoperative data			
Male, *n*(%)	86.5 (32)	65.6 (40)	0.033
Age, year	65.54 ± 10.11	59.36 ± 7.97	0.002
BMI	26.45 ± 2.77	23.98 ± 2.92	<0.001
Hypertension, *n* (%)	54.1 (20)	50.8 (31)	NS
Diabetes mellitus, *n* (%)	24.3 (9)	14.8 (9)	NS
Myocardial infarction, *n* (%)	10.8 (4)	6.6 (4)	NS
Cerebral infarction, *n* (%)	5.4 (2)	8.2 (5)	NS
Renal insufficiency, *n* (%)	10.8 (4)	6.6 (4)	NS
Cigarette smoking, *n* (%)	35.1 (13)	41.0 (25)	NS
Statins, *n* (%)	48.6 (18)	34.4 (21)	NS
LVEF, %	56.78 ± 9.99	56.51 ± 8.23	NS
Pulmonary hypertension, *n* (%)	16.2 (6)	16.4 (10)	NS
Serum TC, mmol/l	4.64 ± 1.05	4.14 ± 1.05	0.016
Serum TG, mmol/l	2.27 ± 1.88	2.25 ± 2.13	NS
Operative procedures			
Number of graftings, n	2.70 ± 0.94	2.08 ± 0.726	0.001
CPB time, min	103.73 ± 37.01	79.26 ± 28.10	<0.001
Aortic clamping time, min	64.05 ± 23.31	51.73 ± 64.05	0.01
Fluid balance, ml	2605.81 ± 1235.09	2550.82 ± 771.55	NS
Postoperative data			
Volume of transfusion, ml	729.73 ± 790.52	277.05 ± 382.16	<0.001
Serum TC, mmol/l	2.74 ± 0.061	2.91 ± 0.87	NS
Serum TG, mmol/l	1.48 ± 1.21	1.20 ± 0.69	NS
FFA, mmol/l	0.87 ± 0.28	0.66 ± 0.22	<0.001
Outcome			
Incubation time, hour	24.84 ± 15.52	21.61 ± 15.09	NS
ICU time, day	3.65 ± 2.50	2.59 ± 2.59	0.027
Hospital time, day	19.92 ± 3.28	18.46 ± 7.11	NS
Mortality, *n* (%)	2.7 (1)	0 (0)	NS

*NS* not significant, *BMI* body mass index, *LVEF* left ventricular ejection fraction, *CPB* cardiopulmonary bypass, *TC* total cholesterol, *TG* triglycerides, *FFA* free fatty acid, *ICU* intensive care unit

### Risk factors for early postoperative hypoxemia

According to Table 1, the following parameters with significant difference were selected as covariates for the logistic regression model: male, age, BMI, preoperative serum TC levels, CPB time, aortic clamping time, number of graftings, volume of transfusion, and postoperative serum FFA concentrations. As shown in Table 2, multivariate stepwise forward logistic regression analysis confirmed that age, BMI and postoperative serum FFA concentrations were independently associated with early postoperative hypoxemia following CABG. Specifically, A 1 mmol/l increase in serum FFA level was related to an odd ratio (OR) of 2.523 [95 % confidence interval (CI), 1.094–14.477, *P* = 0.002].

**Table 2:** Logistic regression of risk factors of patients with early postoperative hypoxemia

	*P* value	OR	95 % CI
Age	0.042	2.696	1.138 ~ 7.146
BMI	0.012	1.52	1.098 ~ 2.103
Postoperative serum FFA levels	0.002	2.523	1.094 ~ 14.477

*FFA* free fatty acid, *OR* odd ratio, *CI* confidence interval

### Changes of perioperative FFA levels and the correlations with TG and the lowest PaO_2_/FiO_2_

**Fig. 1:**
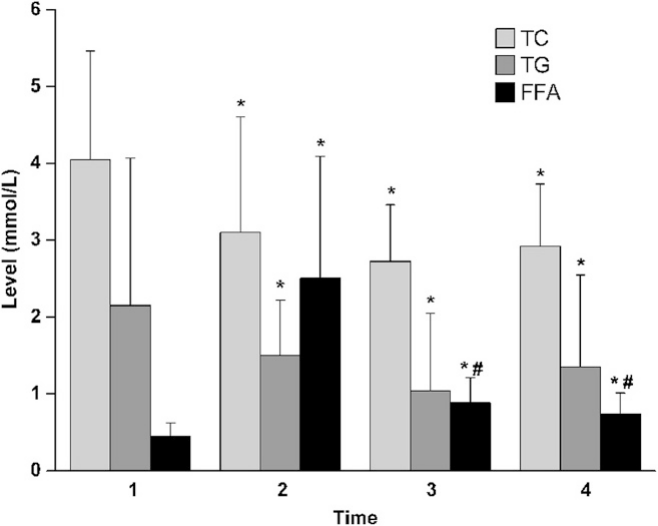
Serum or plasma levels of total cholesterol (TC), triglycerides (TG) and free fat acid (FFA) at four time points. Time point 1, preoperation; Time point 2, 10 min after heparinization; Time point 3, after protamine neutralization; and Time point 4, 2 h postoperation. *P < 0.05 *vs.* Time point 1; #P < 0.05 *vs.* Time point 2

Plasma concentrations of TC and TG decreased significantly after CPB, reaching to their lowest levels when CPB was terminated using protamine. However, both TC and TG levels increased slightly at 2 h after CABG but were dramatically lower than those before CPB (*P* < 0.05). FFA levels elevated rapidly to 2–5 folds after heparizination, whereas reduced gradually during and after CABG and were significantly higher than those before CABG (*P* < 0.05) (Fig. 1).

**Fig. 2:**
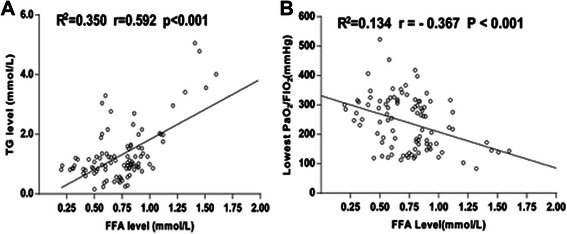
Correlation analyses of serum free fat acid (FFA) concentrations with serum triglycerides (TG) levels (**a**) and lowest PaO_2_/FiO_2_ (**b**)

Polynomial regression analysis demonstrated that postoperative serum TG levels had positive correlation with the postoperative FFA (R [2] = 0.350,r = 0.592, *P* < 0.001) (Fig.2a), and postoperative FFA inversely related to the lowest PaO_2_/FiO_2_ within 24 h after CABG (R [2] = 0.134,r = − 0.367, *P* < 0.001) (Fig.2b).

### Serum concentrations of inflammatory cytokines and their correlation with FFA levels

**Fig. 3:**
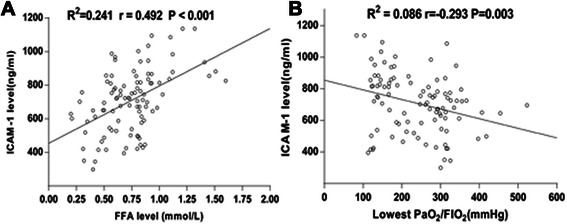
Correlation analyses of serum concentrations of intercellular adhesion molecule-1 (ICAM-1) with serum free fatty acid (FFA) levels (**a**) and lowest PaO_2_/FiO_2_ (**b**)

According to Table 3, patients in the hypoxemia group had markedly higher serum ICAM-1 levels than those in the non-hypoxemia group (799.26 ± 198.30 *vs.* 652.66 ± 159.00 ng/ml, *P* < 0.001), whereas there was no significant difference in serum concentrations of IL-6, TNF and ET-1 between the two groups. In addition, serum FFA levels at 2 h after CABG correlated positively with ICAM-1 levels (R [2] = 0.241,r = 0.492, *P* < 0.001) (Fig. 3a), but had no correlation with IL-6, TNF and ET-1 levels. furthermore, the lowest PaO_2_/FiO_2_ had a negative correlation with serum ICAM-1 concentrations (R [2] = 0.086,r = − 0.293,*P* = 0.03) (Fig. 3b), but no association with IL-6, TNF and ET-1 levels.

**Table 3:** Inflammatory parameters of the postoperative patients

	Hypoxemia group	Non-hypoxemia group	*P* value
	(*n* = 37)	(*n* = 61)	
IL-6 (pg/ml)	88.22 ± 69.55	72.12 ± 45.30	NS
TNF (pg/ml)	56.72 ± 35.12	67.15 ± 57.86	NS
ET-1 (pg/ml)	10.73 ± 6.78	10.83 ± 5.67	NS
ICAM-1 (ng/ml)	799.26 ± 198.30	652.66 ± 159.00	<0.001

*IL-6* interleukin-6, *TNF-α* tumor necrosis factor-α, *ET-1* endothelin-1, *ICAMn1* intercellular adhesion molecule-1

## Discussion

The description of predisposing factors for postoperative pulmonary dysfunction can help future identification of high-risk patients before surgery. Manipulation of potential factors or implementation of novel therapy to attenuate or prevent pulmonary injury can be applied in a selected group of high risk patients. Nevertheless, the respiratory failure in patients with cardiac surgery could not be diagnosed technically as ARDS, because it was not usually accompanied by typical radiological pulmonary infiltrates. Therefore, previous research varied the definition of hypoxemia [3,12–17]. Several studies focused on early period. Rady et al. [15] reported 12 % incidence of early hypoxemia (PaO_2_/FiO_2_ ≤ 150 mm Hg at the time of admission to the cardiovascular ICU). Weiss et al. [17] suggested that hypoxemia (PaO_2_/FiO_2_ < 300 mm Hg) within first 12 h happened in only 1.32 % patients, and had no effect on clinical outcome. In the present study, early postoperative hypoxemia was defined as PaO_2_/FiO_2_ ≤ 200 mm Hg in the first 24 h after cardiac surgery. In this study, the incidence rate of postoperative hypoxemia was 37.8 %, which surpassed markedly the 27.8 % found in another report [18].

Recent studies have shown that the main risk factors for hypoxemia after cardiac surgery are age, BMI, prolonged CPB time, smoking, reduced cardiac function, hypoalbuminemia, previous myocardial infarction, and history of cerebral vascular disease [14–18], In this study, multiple logistic regression analysis revealed that age, BMI and postoperative serum FFA levels were independently associated with early postoperative hypoxemia in patients undergone on-pump CABG. The present study for the first time demonstrated that serum FFA levels were significantly higher in the hypoxemia group than in the non-hypoxemia group, and that elevated FFA concentration was a risk factor for early postoperative hypoxemia after on-pump CABG, which may be closely associated with lung injury secondary to endothelial activation.

Some research delineated that advanced age and BMI were risk factors related to hypoxemia after CABG [14–17]. Each year after age 34 increased the risk by 0.32 % [16]. Akdur *et al.* [12] found that obesity had a detrimental effect on blood gas measurements, pulmonary function, exercise capacity and complications rates in postoperative period following CABG surgery. With resemblance to the above-mentioned results, the present study also verified that age and BMI were independent risk factors for early postoperative hypoxemia.

In addition, other studies indicated that CPB was also the important cause of lung injury [1–3,13]. CPB initiates a profound systemic inflammatory cascade that can lead to pulmonary injury. Moreover, there was pulmonary ischemia-reperfusion (IR) injury occurred during and after CPB [1,2]. CPB increased intrapulmonary shunt and atelectasis, impaired the oxygenation [13]. CPB time was a risk factor of postoperative pulmonary insufficiency [3,15,17]. However, in this study, although CPB time was longer in the hypoxemia group than in the non-hypoxemia group through univariate analysis, it was not a risk factor for hypoxemia in multivariate regression analysis. This result may be related to lower pump time (less than 120 min) in 80 % of patients, which was similar to the finding by Santos et al. [14]. However, little was known about the role of FFA in lung injury associated with CPB. FFA derives from the decomposition of TG. It was indicated that FFA could result in lung injury secondary to lung fat embolism [19]. Additionally, oleic acid, an eighteen-carbon monounsaturated fatty acid, has been used to make the animal model of acute lung injury [19]. The present study demonstrated that elevated serum FFA concentration was positively associated with an increase in TG level. FFA levels elevated markedly after heparinization, whereas decreased gradually during and after CABG and were significantly higher than those before CABG. Of note, patients with early postoperative hypoxemia had significantly higher FFA levels in comparison to those without hypoxemia. Furthermore, elevated serum FFA levels correlated inversely with lowest PaO_2_/FiO_2_ in 24 h after CABG. Multiple logistic regression analysis suggested that FFA was an independent risk factor for early postoperative hypoxemia.

There were a variety of mechanisms whereby FFA induced lung injury. Vadasz et al. [20] revealed that oleic acid could suppresses both amiloride-sensitive sodium channels and the Na^+^, K^+^-ATPase, and thus promote alveolar edema formation as well as prevent edema resolution, thereby contributing to the development of ARDS. Golbidi et al. [21] showed that oleic acid could reduce the intracellular ATP level and increase vascular permeability, thus leading to pulmonary edema. Recently, several studies have assessed the role of FFA as a trigger for endothelial activation, inflammation and thrombosis. Moreover, a clinical experiment conducted by Mathew et al. [22] found that lipid infusion could increase significantly plasma FFA concentration, and that mean plasma FFA levels correlated closely with endothelial activation markers: ICAM-1 and other adhesion molecules. It is well known that ICAM-1 is one of adhesion molecules which are largely found on endothelium, and that an increase in soluble ICAM-1 level resulted either from increased expression in activated endothelial cells or from fortified proteolytic cleavage of endothelium bound forms secondary to endothelial cell injury [23]. The present study confirmed that increased FFA concentration after CPB had a significantly positive correlation with serum ICAM-1 level, which perhaps indicated that FFA could induce endothelial activation and damage.

Additionally, Görlach et al. reported that respiratory insufficiency after CPB was associated with a distinct increase in the ICAM-1 [24]. A small study of pediatric cardiac surgery indicated that there was a significant inverse relationship between plasma levels of ICAM-1 and PaO_2_/FiO_2_ ratio [25]. Similarly, the current study also demonstrated that serum ICAM-1 levels were inversely related to lowest PaO_2_/FiO_2_, suggesting that endothelial dysfunction may be the reason of lung injury. Further, ICAM-1 was thought to play an important role in neutrophil recruitment and trafficking into the lung [26]. An elevated expression of ICAM-1 in the lung was associated with an increased accumulation of neutrophils in a canine model of CPB [27]. Once adherent to the endothelium, neutrophils release cytotoxic proteases and oxygen-derived free radicals, which are responsible for the lung edema and oxygenation impairment [28].

Briefly, CPB-induced systemic inflammatory response remains extremely complex and far from being fully understood. Previous research verified elevated levels of TNF-α, IL-6 and ET-1 were associated with oxygenation impairment during CPB [29,30]. However, we did not find any relationship between one of those cytokines and hypoxemia.

This study has several limitations. First, the patient population was relatively small. Second, the overall impact of different kinds of fatty acids on the vascular bed had not been clearly clarified. Third, as we excluded patients with obvious organ dysfunction and emergency surgery, the potential selective bias could not be completely avoided.

## Conclusion

Serum FFA levels played an important part in the development of postoperative hypoxemia after cardiac surgery. Elevated FFA concentration was a risk factor for early postoperative hypoxemia after CABG with CPB and may induce lung injury via endothelial activation.

## References

[1] Apostolakis E, Filos KS, Koletsis E, Dougenis D (2010). Lung dysfunction following cardiopulmonary bypass. J Card Surg.

[2] Stephens RS, Shah AS, Whitman GJ (2013). Lung injury and acute respiratory distress syndrome after cardiac surgery. Ann Thorac Surg.

[3] Wang Y, Xue S, Zhu H (2013). Risk factors for postoperative hypoxemia in patients undergoing Stanford A aortic dissection surgery. J Cardiothorac Surg.

[4] Mehta A, Oeser AM, Carlson MG (1998). Rapid quantitation of free fatty acids in human plasma by high-performance liquid chromatography. J Chromatogr B Biomed Sci Appl.

[5] Van Veen JJ, Laidlaw S, Swanevelder J, Harvey N, Watson C, Kitchen S (2009). Contact factor deficiencies and cardiopulmonary bypass surgery: detection of the defect and monitoring of heparin. Eur J Haematol.

[6] Brunner MP, Shah SH, Craig DM, Stevens RD, Muehlbauer MJ, Bain JR (2011). Effect of heparin administration on metabolomic profiles in samples obtained during cardiac catheterization. Circ Cardiovasc Genet.

[7] Kimura T, Toung J, Margolis S, Bell W, Cameron J (1980). Respiratory failure in acute pancreatitis: the role of free fatty acids. Surgery.

[8] Azekoshi Y, Yasu T, Watanabe S, Tagawa T, Abe S, Yamakawa K (2010). Free fatty acid causes leukocyte activation and resultant endothelial dysfunction through enhanced angiotensin II production in mononuclear and polymorphonuclear cells. Hypertension.

[9] Peltier LF (1988). Fat embolism. A perspective. Clin Orthop Relat Res.

[10] Bursten SL, Federighi DA, Parsons P, Harris WE, Abraham E, Moore EE (1996). An increase in serum C18 unsaturated free fatty acids as a predictor of the development of acute respiratory distress syndrome. Crit Care Med.

[11] de Vries AJ, Gu YJ, van Oeveren W (2002). The rationale for fat filtration during cardiac surgery. Perfusion.

[12] Akdur H, Yigit Z, Sozen AB, Cagatay T, Guven O (2006). Comparison of pre- and postoperative pulmonary function in obese and non-obese female patients undergoing coronary artery bypass graft surgery. Respirology.

[13] Andrejaitiene J, Sirvinskas E, Bolys R (2004). The influence of cardiopulmonary bypass on respiratory dysfunction in early postoperative period. Medicina (Kaunas).

[14] dos Santos NP, Mitsunaga RM, Borges DL, Costa Mde A, Baldez TE, Lima IM (2013). Factors associated to hypoxemia in patients undergoing coronary artery bypass grafting. Rev Bras Cir Cardiovasc.

[15] Rady MY, Ryan T, Starr NJ (1997). Early onset of acute pulmonary dysfunction after cardiovascular surgery: risk factors and clinical outcome. Crit Care Med.

[16] Szeles TF, Yoshinaga EM, Alenca W, Brudniewski M, Ferreira FS, Auler JO (2008). Hypoxemia after myocardial revascularization: analysis of risk factors. Rev Bras Anestesiol.

[17] Weiss YG, Merin G, Koganov E, Ribo A, Oppenheim-Eden A, Medalion B (2000). Postcardiopulmonary bypass hypoxemia: a prospective study on incidence, risk factors, and clinical significance. J Cardiothorac Vasc Anesth.

[18] Ji Q, Mei Y, Wang X, Feng J, Cai J, Sun Y (2008). Study on the risk factors of postoperative hypoxemia in patients undergoing coronary artery bypass grafting. Circulation J.

[19] Wang HM, Bodenstein M, Markstaller K (2008). Overview of the pathology of three widely used animal models of acute lung injury. Eur Surg Res.

[20] Vadasz I, Morty RE, Kohstall MG, Olschewski A, Grimminger F, Seeger W (2005). Oleic acid inhibits alveolar fluid reabsorption: a role in acute respiratory distress syndrome?. Am J Respir Crit Care Med.

[21] Golbidi S, Moriuchi H, Yang C, Irikura M, Irie T, Hamasaki N (2003). Preventive effect of phosphoenolpyruvate on hypoxemia induced by oleic acid in Guinea pigs. Biol Pharm Bull.

[22] Mathew M, Tay E, Cusi K (2010). Elevated plasma free fatty acids increase cardiovascular risk by inducing plasma biomarkers of endothelial activation, myeloperoxidase and PAI-1 in healthy subjects. Cardiovasc Diabetol.

[23] Balciunas M, Bagdonaite L, Samalavicius R, Baublys A (2009). Markers of endothelial dysfunction after cardiac surgery: soluble forms of vascular-1 and intercellular-1 adhesion molecules. Medicina (Kaunas).

[24] Gorlach G, Sroka J, Heidt M, Knez I, Sablotzki A, Schonburg M (2003). Intracellular adhesion molecule-1 in patients developing pulmonary insufficiency after cardiopulmonary bypass. Thorac Cardiovasc Surg.

[25] Boldt J, Osmer C, Linke LC, Dapper F, Hempelmann G (1995). Circulating adhesion molecules in pediatric cardiac surgery. Anesth Analg.

[26] Calfee CS, Eisner MD, Parsons PE, Thompson BT, Conner ER, Matthay MA (2009). Soluble intercellular adhesion molecule-1 and clinical outcomes in patients with acute lung injury. Intensive Care Med.

[27] Dreyer WJ, Burns AR, Phillips SC, Lindsey ML, Jackson P, Kukielka GL (1998). Intercellular adhesion molecule-1 regulation in the canine lung after cardiopulmonary bypass. J Thorac Cardiovasc Surg.

[28] Boyle EM, Pohlman TH, Cornejo CJ, Verrier ED (1996). Endothelial cell injury in cardiovascular surgery: ischemia-reperfusion. Ann Thorac Surg.

[29] Duffy JY, Schwartz SM, Lyons JM, Bell JH, Wagner CJ, Zingarelli B (2005). Calpain inhibition decreases endothelin-1 levels and pulmonary hypertension after cardiopulmonary bypass with deep hypothermic circulatory arrest. Crit Care Med.

[30] Mahle WT, Matthews E, Kanter KR, Kogon BE, Hamrick SE, Strickland MJ (2014). Inflammatory response after neonatal cardiac surgery and its relationship to clinical outcomes. Ann Thorac Surg.

